# Fibre Bragg Gratings for the Monitoring of Wooden Structures

**DOI:** 10.3390/ma11010007

**Published:** 2017-12-21

**Authors:** Roberto Marsili, Gianluca Rossi, Emanuela Speranzini

**Affiliations:** Department of Engineering, University of Perugia, via Duranti, 93-06125 Perugia, Italy; gianluca.rossi@unipg.it (G.R.); emanuela.speranzini@unipg.it (E.S.)

**Keywords:** optical fibre, FBG sensor, wood, strain analysis, experimental analysis, monitoring

## Abstract

The aim of this work was to develop and validate an experimental methodology suitable for analysing on-site the behaviour of fibre-reinforced wooden structures. The proposed measurement method is based on the application of fibre Bragg grating (FBG) strain sensors. An analysis of adhesive behaviour was performed preliminarily, which provided indications for choosing the type of adhesive and for the fibre bonding length in accordance with the volume of measurement. The first series of tests was carried out on wood samples to verify the coupling between the measuring sensor and the wood support when the latter is subject to mechanical stresses. The second investigation was done on site to test the behaviour of a historical wood floor before and after reinforcement by means of a series of tests performed using optical fibres with the Bragg grating. The optical fibre system measurements were compared to those obtained using a laser vibrometer, a measurement system of proven stability and precision. The comparison makes it possible to confirm the validity of the results and the reliability of the system for the monitoring of historic wooden structures.

## 1. Introduction

Optical fibre is a device that can act as a waveguide for light radiation. When transmitting signals, its optical properties remain constant along the entire length of the fibre. If some optical properties are modified in a limited portion of the waveguide through modulation of the refractive index, a sensor can be obtained, the so-called Bragg grating sensor, which is sensitive to mechanical stresses and therefore is able to measure the deformations in the structure to which it is applied.

In the last several years, the polymer optical fibre sensors have been frequently employed for civil aircraft, structural health monitoring, healthcare and biomedicine fields. The properties of polymers have been explored to identify situations offering potential advantages over conventional silica fibre sensing technology, replacing, in some cases, problematic electronic technology used in these mentioned fields, where there are some issues to overcome [1]. The Fibre Bragg gratings (FBG) can be used as thermomechanical sensors by means of the annealing process, which decreases the residual birefringence to a lower extent as well [2]. In [3], a cost effective solution to produce optical fibre relative humidity sensors based on Fabry–Perot interferometer micro-cavities is proposed. The device is manufactured by the recycling of optical fibres destroyed through the catastrophic fuse effect, which considerably reduces the manufacturing costs. The micro-cavities were filled with an organo-silica hybrid material, called di-ureasil, allowing the sensing of relative humidity. A novel and highly sensitive liquid level sensor based on a polymer optical fibre Bragg grating (POFBG) is experimentally demonstrated. In [4], two different configurations are studied and both configurations show the potential to interrogate at a liquid level. The FBG are also widely used as pressure and temperature sensors. Indeed, Bragg gratings photo-inscribed in polymer optical fibres (POFs) are more sensitive to temperature and pressure than their silica counterparts because of their larger thermo-optic coefficient and smaller Young’s modulus [5,6]. In chemistry, by a tilted fibre Bragg grating sensor, you can investigate individual detection of different anion concentrations [7]. A silica fibre Bragg grating has the potential to be applied in tunable optical filters and tunable cavities for photonic application. A silica fibre Bragg grating has the potential to be applied on tunable optical filters and tunable cavities for photonic application [8].

Thanks to their particular metrological performance, these sensors can be used for health monitoring of structures. FBG sensors are very small instruments, thus they are non-invasive and can be embedded in materials or inserted in very small spaces. They are characterized by high stability [9] due to the material (glass) from which the optical fibre is made, are immune to radiation and resistant to aging, corrosion and atmospheric agents [10], and can be used for years without requiring calibration. They provide accurate results and good signal transmission over long distances. 

Applications in bridges and other civil structures have also been proposed by many authors, for example in Japan [11,12,13]. The first applications in Italy regard the monitoring of bridges in reinforced concrete, and later they were used for the first time for the monitoring of historic masonry buildings [14]. Because of their flexibility and small size, optical fibres can be glued directly to the structure without modifying it and without compromising aesthetics. All that protrudes from the structure are the connectors needed for connection to the acquisition system. Optical fibre systems are easily adaptable for the monitoring of structures reinforced with Fibre Reinforced Polymer (FRP) composite materials [15,16] because they can be applied directly to the reinforcing fibres, as opposed to other non-contact measurement methods that are affected by numerous interference inputs [17,18,19]. When many measurement points are necessary, they can also become a low-cost technique (each one costs some ten euros) compared to the classical electrical strain gauges. In this case, the fibre’s multiplexing capability makes it possible to have many measurement points on a single fibre, thereby also reducing costs due to sensor connections, installation and signal transmission [20,21,22,23,24,25,26]. The strain field transferred from the structure to the optical fibre sensor generates changes in the characteristics of the light signal transmitted by the glass core of the optical fibre. However, the mechanical properties of the protected coatings employed in conjunction with optical fibre alter the strain transduction capabilities of the sensor. A portion of the strain is absorbed by the protective coating of the optical fibre, and, hence, only a segment of structural strain is sensed. In [27], a model is introduced and tested through which it is possible to interpret the actual level of structural strains from the values measured by an optical fibre sensor [28,29,30]. An analytical model for the relationship between the strain measured by a fibre Bragg grating sensor and the actual structural strain is in [31]. Other studies regarding the light transmission ability of reinforcing glass fibres used in polymer composites were carried out in [32]. Few efforts were turned to the use of FBG in wood structures. Interesting research is presented in [33], where the wavelength evolution measured by FBG sensors was used to define the fracture process zone development phase and, in [34], the use of a FBG sensor, in situ measurement and continuous monitoring of deformations in painted wood panels were proposed. Other research regards stereovision measurements to evaluate the modulus of elasticity of wood by compression tests parallel to the grain [35], and studies on the variation of transverse and shear stiffness properties of wood [36] and on the wood quality evaluation by spatial variation of elastic properties within the stem [37].

This work proposes the use of FBG sensors to monitor wooden beams in operating conditions and to analyse the effects of the structural reinforcement done to them. In fact, in the last decade, many studies have been done on the reinforcement of wooden structures, such as natural fibre reinforcement beams [38,39] and reversible reinforcement with unbounded composite plates [40]. During the operations for the reinforcement of historical structures, the need arose to measure the carrying capacity of the reinforced structures over time to assess the goodness of the intervention [41,42].

In this research, an analysis of adhesive behaviour was performed preliminarily, which provided indications for choosing the type of adhesive and for the fibre bonding length. The study consists of two phases: a first series of measurements made on small wooden samples to test the quality of the coupling between the measuring sensor and the wood; and a second on-site test aimed at verifying and validating the use of FBGs for monitoring the bending behaviour of reinforced wood beams. The verification of the coupling between sensors and wood was due to the lack of data in the literature, whereas these studies are of fundamental importance for the evaluation of the proposed applications [43,44]. Particular significance must be given in fact to the characteristics of the support and to the way in which the sensors are applied [45]. Furthermore, as they are applied by gluing, the measurements recorded could be affected by the characteristics of the adhesive, and given that wood is not a uniform material, and may have a rough surface, there is the risk of obtaining measurements of unsatisfactory precision. On-site tests were carried out by measuring the response of the structures subjected to impulsive loads. The structural response was simultaneously measured by the FBG sensors installed on the structures and by a laser Doppler vibrometer, as it is better to have dynamic information than methods such as digital image correlation [46,47,48]. The positive result of the comparison assures the reliability of the proposed measurement technique in the monitoring of these structures.

## 2. Optical Fibre and Bragg Sensors

The manufacturing process of the sensing element is based on the photorefractive effect, which is a phenomenon whereby a material illuminated by electromagnetic radiation at certain wavelengths causes a permanent modification of its refractive index. Fibre Bragg grating sensors can be built with a periodic modulation of the refraction index of a single mode silica fibre on a Ge-doped segment [49]. 

**Figure 1:**
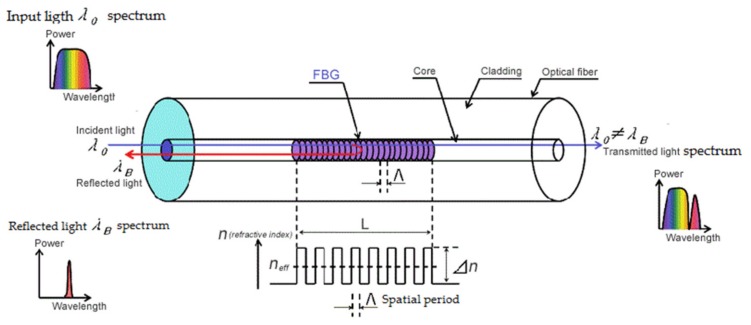
Principle of measurement of the FBG sensors.

Once the grating is inscribed on the fibre, a broad band light source is coupled at one end of the fibre. As is shown in Figure 1 some light is back-reflected. The central wavelength of the reflected spectrum, defined as Bragg’s wavelength *λ_B_*, is expressed by the following relationship:

(1) $$\lambda_{B}=2\cdot n_{eff}\cdot \Lambda$$

where *n_eff_* is the effective refraction index of the fibre core and Λ is the value of the modulation step.

When the sensitive fibre segment (the grating) is deformed, a change takes place in the modulation step Λ, and consequently the output Bragg wavelength changes according to Equation (1). Since the initial period of the grating is a known parameter, analysing the shift in the wavelength reflected can be traced back to the deformation applied to the grating. The strain of the sensitive fibre segment should be equal to the strain of the structure to which it is glued. Therefore, the strain transfer mechanism across various different interfaces, such as material-adhesive, adhesive-jacket and jacket-fibre core, must be carefully analysed.

The tests of this work were conducted using FBG sensors type OS1100 provided by Micron Optics (Athanta, GA, USA). Their characteristics are indicated in Table 1.

### Model for Bare Optical Fibre Inserted inside the Wooden Structure

**Figure 2:**
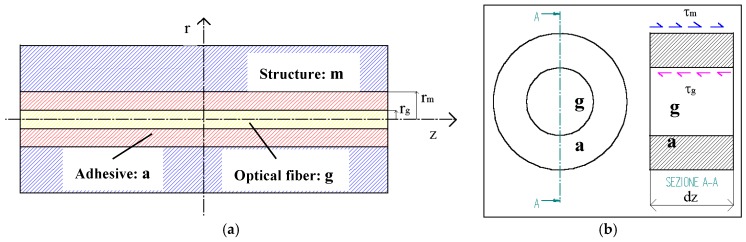
(**a**) Longitudinal section of the optical fibre; (**b**) transversal section.

In order to better evaluate the uncertainty of the measurement result due to the effect of the adhesive, an analysis of the mechanism of the strain transfer to the optical fibre was performed, with reference to the classical theory and it was specifically carried out for the wooden structures in the situation foreseen in this study, i.e., fibre without coating, and bonding with bi-component epoxy adhesive. It provided indications for choosing the type of adhesive and for the optical fibre bonding length. The following hypotheses are assumed: (1) the optical fibre is without coating; (2) the load is applied in parallel to the fibre; (3) the thermal variations are negligible compared to the other loads acting on it; (4) all the materials have linear-elastic, homogeneous and isotropic behaviour; (5) the bond is perfect and there is no debonding in the interface between the materials; and (6) the cylindrical symmetry is considered. The optical fibre configuration is shown in Figure 2a (longitudinal section, cutting through a plane containing the fibre axis) and Figure 2b (transversal section). 

As a consequence, this phenomenon can be analysed in the plane (*z*, *r*) and Hooke’s law can be considered; thus, the displacements on the interface are:

*u_m_*(*z*, *r*) = *u_a_*(*z*, *r*) *forr* = *r_m_andu_a_*(*z*, *r*) = *u_g_*(*z*, *r*) *forr* = *r_g_* (2)

the strain of the optical fibre *ε_g_* depends on the variable *z*, exclusively:

*ε_g_*(*z*, *r*) ≡ *ε_g_*(*z*), (3)

and compatibility condition in the section of the fibre *z* = 0:

*ε_m_*(0, *r*) = *ε_a_*(0, *r*) = *ε_g_*(0, *r*), (4)

where *ε_m_*, *ε_a_*, *ε_g_* are the strain in host material, adhesive and optical fibre, respectively.

Assume that the structure is subjected to a tensile axial load N applied at two ends symmetrically. The analysis begins by writing the equilibrium in the *z*-direction of the forces acting on the infinitesimal element (Figure 2b), which gives:

(5) $$-\tau_{g}\cdot dz\cdot 2\cdot \pi \cdot r_{g}+\tau_{m}\cdot dz\cdot 2\cdot \pi \cdot r_{m}=0,$$

from which it is possible to obtain

(6) $$\tau_{m}=\tau_{g}\cdot \frac{r_{g}}{r_{m}}\;\mathrm{or}\;\tau_{g}=\tau_{m}\cdot \frac{r_{m}}{r_{g}},$$

and, considering the generic variable *r*∊[*r_g_*, *r_m_*], the following relationships are obtained:

(7) $$\tau \left(z,r\right)=\frac{r_{g}}{r}\cdot \tau_{g}\left(z\right)\;\mathrm{or}\;\tau \left(z,r\right)=\frac{r_{m}}{r}\cdot \tau_{m}\left(z\right).$$

The axial displacements of the fibre and of the structure are calculated by integrating, on the *z*-axis, the strains *ε_g_* and *ε_m_* and using Hooke’s law. The result is the following:

(8) $$u_{g}(z)=\int_{0}^{z}\frac{\sigma_{g}(z)}{E_{g}}\cdot dz\;\;u_{m}(z)=\int_{0}^{z}\frac{\sigma_{m}(z)}{E_{m}}\cdot dz$$

where *σ_g_*, *E_g_*, *σ_m_*, *E_m_* are stress and Young’s modulus in the optical fibre and host material, respectively.

**Figure 3:**
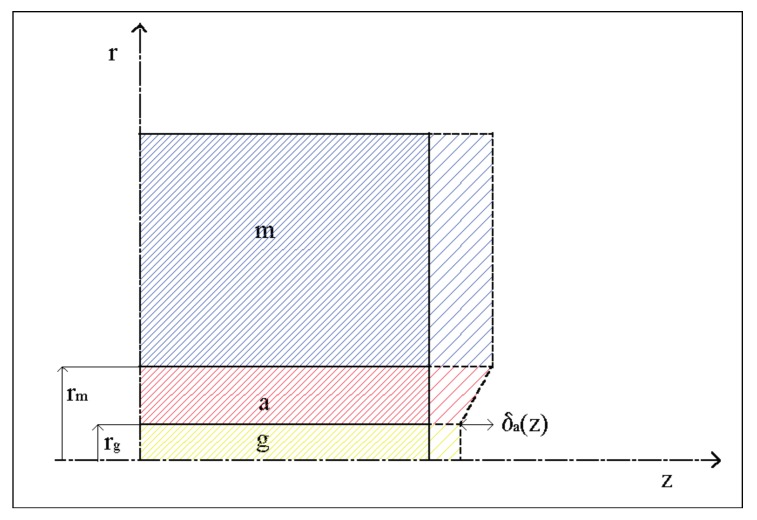
Angular distortion of the adhesive due to the tangential stresses.

In addition, the angular distortion of the adhesive due to the tangential stresses is considered (Figure 3), so that the displacements of the structure can be obtained:

(9) $$u_{m}\left(z\right)=u_{g}\left(z\right)+\delta_{a}\left(z\right)\;\mathrm{with}\;\delta_{a}(z)=\frac{1}{G_{a}}\cdot \int_{r_{g}}^{r_{m}}\tau (z,r)\cdot dr,$$

with *G_a_* being the adhesive shear modulus.

Substituting Equation (8) for Equation (9), the following is obtained:

(10) $$\int_{0}^{z}\frac{\sigma_{m}\left(z\right)}{E_{m}}\cdot dz=\int_{0}^{z}\frac{\sigma_{g}\left(z\right)}{E_{g}}\cdot dz+\frac{1}{G_{a}}\cdot \int_{r_{g}}^{r_{m}}\tau \left(z,r\right)\cdot dr,$$

and, substituting $\sigma_{g}\left(z\right)=\frac{N_{g}\left(z\right)}{\pi \cdot r_{g}{}^{2}}$, Equation (10) becomes:

(11) $$\int_{0}^{z}\frac{\sigma_{m}\left(z\right)}{E_{m}}\cdot dz=\frac{1}{\pi \cdot r_{g}{}^{2}\cdot E_{g}}\cdot \int_{0}^{z}N_{g}\left(z\right)\cdot dz+\frac{1}{G_{a}}\cdot \int_{r_{g}}^{r_{m}}\tau \left(z,r\right)\cdot dr,$$

where *N_g_* is the fibre axial force: $N_{g}\left(z\right)=\pi \cdot r_{g}{}^{2}\cdot \sigma_{g}-2\cdot \pi \cdot r_{m}\cdot \int_{0}^{z}\tau \left(z,r_{m}\right)\cdot dz$, and *σ_g_* is the fibre axial stress in the central section (*z* = 0).

By deriving Equation (11) with respect to *z*, a second order differential equation, a function of *τ_g_*(*z*), is obtained. This differential equation is solved, making reference to the boundary conditions in sections of the fibre *z* = 0 and *z* = *L_f_*, with *L_f_* being the fibre bonding length:

(12) $$z\;=\;0\;N_{g}\left(0\right)=\pi \cdot r_{g}{}^{2}\cdot \sigma_{g}\;\Rightarrow \;N_{g}\left(0\right)=\pi \cdot r_{g}{}^{2}\cdot \sigma_{o}\cdot \frac{E_{g}}{E_{m}},$$

(13) $$z=L_{f}\;N_{g}\left(L_{f}\right)=0,$$

which gives the result:

(14) $$\tau_{g}\left(z\right)=\frac{\sigma_{g}\cdot r_{g}\cdot \lambda }{2\cdot senh\left(\lambda \cdot L_{f}\right)}\cdot \cosh\left(\lambda \cdot z\right),$$

with

(15) $$\lambda =\sqrt{\frac{2\cdot G_{a}}{E_{g}\cdot r_{g}{}^{2}\cdot \ln\left(\frac{r_{m}}{r_{g}}\right)}}$$

being a constant that takes into account both the geometry and material characteristics of the system analysed. 

With reference to $\tau \left(z,r\right)=\frac{r_{g}}{r}\cdot \tau_{g}\left(z\right)$ and to $\sigma_{g}\left(z\right)=\frac{N_{g}\left(z\right)}{\pi \cdot r_{g}{}^{2}}$, the following relationship is obtained:

(16) $$\sigma_{g}\left(z\right)=\sigma_{g}-\frac{2}{r_{g}}\cdot \left[\frac{C_{1}}{\lambda }\cdot sinh\left(\lambda \cdot z\right)\right],$$

being

(17) $$C_{1}=\frac{\sigma_{g}\cdot r_{g}\cdot \lambda }{2\cdot \sinh\left(\lambda \cdot L_{f}\right)}\;\text{with}\;L_{f}\;\text{the fibre bonding length}$$

At this point, with reference to $\varepsilon_{g}\left(z\right)=\frac{\sigma_{g}\left(z\right)}{E_{g}}$ and to the compatibility equation $\varepsilon_{g}\left(0\right)=\varepsilon_{m}\left(0\right)=\varepsilon_{0}$, it is possible to obtain the fibre axial strain:

(18) $$\varepsilon_{g}\left(z\right)=\varepsilon_{0}\cdot \left[1-\frac{\mathrm{senh}\left(\lambda \cdot z\right)}{\mathrm{senh}\left(\lambda \cdot L_{f}\right)}\right].$$

Having chosen *L_f_*, Equation (18) represents the link between the deformation detected by the FBG sensor *ε_g_*(*z*) and actual deformation of the structure (*ε*_0_).

The minimum gluing length *L_f_* can be calculated as a function of the maximum difference considered tolerable between the strain on the host material *ε*_0_ and the strain on the fibre core *ε_g_*, defined as:

(19) $$\Delta =\frac{\varepsilon_{0}-\varepsilon_{g}\left(L_{g}\right)}{\varepsilon_{0}}\;\Rightarrow \;\varepsilon_{0}-\varepsilon_{0}\cdot \left[1-\frac{\mathrm{senh}\left(\lambda \cdot L_{g}\right)}{\mathrm{senh}\left(\lambda \cdot L_{f}\right)}\right]=\Delta \cdot \varepsilon_{0},$$

from which:

(20) $$L_{f}=\frac{1}{\lambda }\cdot {\mathrm{senh}}^{-1}\cdot \left[\frac{1}{\Delta }\cdot \mathrm{senh}\left(\lambda \cdot L_{g}\right)\right],$$

with *L_g_* being the FBG sensor length and *L_f_* the fibre bonding length.

**Figure 4:**
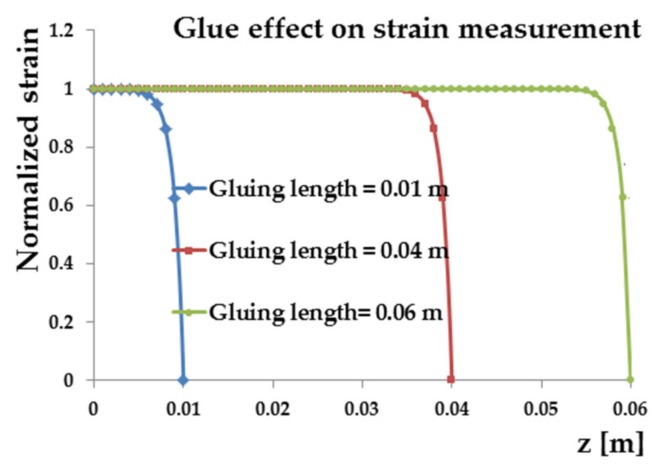
Effects of different gluing lengths.

**Figure 5:**
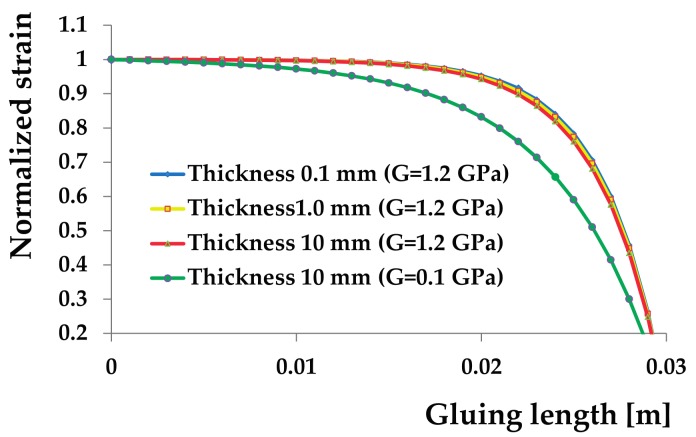
Effects of the glue thickness on the strain measurement.

The normalized strain trend defined as $\varepsilon_{g}\left(z\right)/$($\varepsilon_{0})$ has been plotted for 0.01, 0.04 and 0.06 m bonding length in the diagram shown in Figure 4. The longer the bonding length of the fibre, the greater its sensitivity because there are more Bragg gratings that deform and thus a greater variation of Δ*λ*. The effect of the adhesive thickness has also been studied having fixed the gluing length. In Figure 5, the curves corresponding to the tangential stiffness modulus of 1.2 GPa, a typical value of a common epoxy adhesive, show no effect on the ratio between the strain of the fibre sensor with the strain of the supporting material. On the contrary, significant effects are obtained using adhesives or resins with low values of their tangential module.

From this analysis, it was possible to establish that the measurement uncertainty will fall within an acceptable range when the percentage difference Δ, between *ε_g_* and *ε*_0_, is less than 1%. 

With the results illustrated above, by appropriately choosing glue and gluing length, it is thus possible to reduce within tolerable limits the sources of uncertainty due to the installation effects. Other sources of error can result from the system used to detect Bragg’s wavelength. Here, a Micron Optic FBG measurement system was used, with an uncertainty component due to its resolution equal to 1 µε. Significant changes in temperature can occur during the tests, causing measurement uncertainty. The experimental tests of this research were carried out in controlled temperature laboratory or in unvarying temperature places in which the temperature compensation was not necessary. When it will be needed, for example in outdoor tests with variable weather, the compensation will be performed as widely reported in the literature [50,51].

## 3. Results

### 3.1. Wooden Beam Measurements

The experiment was conducted using FBG sensors type OS1100 provided by Micron Optics (Atlanta, GA, USA). They had bare fibres, i.e., without coating, which has the function of protecting the inner part of the fibre in which the actual propagation of the electromagnetic signal takes place. The absence of the coating protects against measurement errors connected with the sliding of the outer layer from the internal layers. The difficulty of using optical fibres without coating on-site should be noted, however, given their great delicateness and the accidental mechanical stresses to which they may be subjected both during installation and during their service life in buildings.

#### 3.1.1. Verification of FBG Application to Wood

In this stage of the investigation, compression, tensile and shear tests were carried out on fir wood samples prepared and tested in the laboratory. All the samples were instrumented with FBG sensors. Electrical strain gauges were also applied, and were compensated for temperature, in order to compare the results obtained from the two measurement systems.

**Figure 6:**
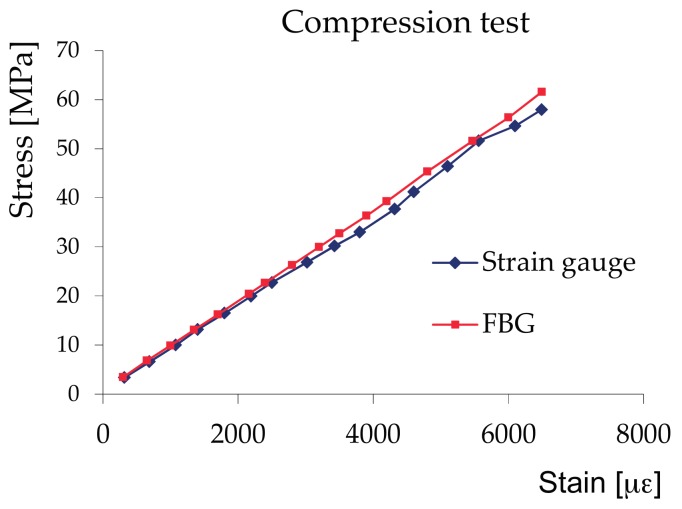
Compression test: stress–strain diagram.

Compression tests—UNI ISO 3261 samples (for the determination of the elasticity modulus), dimensions 50 × 50 × 220 mm. FBG and strain gauges were glued on the same face of the specimen and they covered a little larger surface than the size of the growth rings. The load was generated through an Instron tester machine (Instron, Norwood, MA, USA) at a fixed displacement rate of 2.0 mm/min. The tests showed a good correspondence (Figure 6) between the deformations measured by the FBG compared to those measured by the strain gauges, although sometimes there were divergences for values close to 2/3 of the failure load in cases where the fracture line is transverse to the line of application of the strain gauge or of the optical fibre. The maximum deviation between the measurements with FBG and that with strain gauges is equal to 5%.

**Figure 7:**
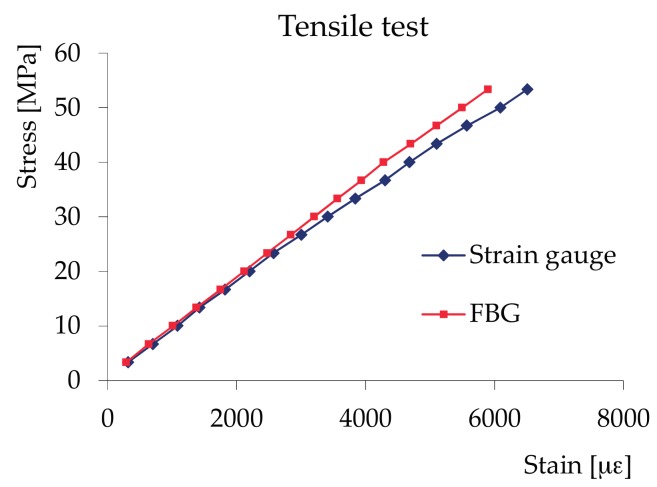
Tensile test: stress–strain diagram.

Tensile tests—Tensile tests-UNI ISO 3345 samples (for the determination of the resistance parallel to the grain), dimensions 100 × 390 × 20 mm. The load was generated through an Instron tester machine at a fixed displacement rate of 2.0 mm/min. FBG and strain gauges were glued on the same face of the specimen. As shown in Figure 7, the deformations measured with the two techniques were a perfect match until a stress close to 40 MPa. The cause of the sudden changes can be seen in distortions of the Fibre Bragg Gratings (FBG) spectrum. The limited robustness of the fibre-optic sensors against damage and difficulties with fibre handling are important application problems that have been highlighted in the technical literature, as, for example, in [52,53].

**Figure 8:**
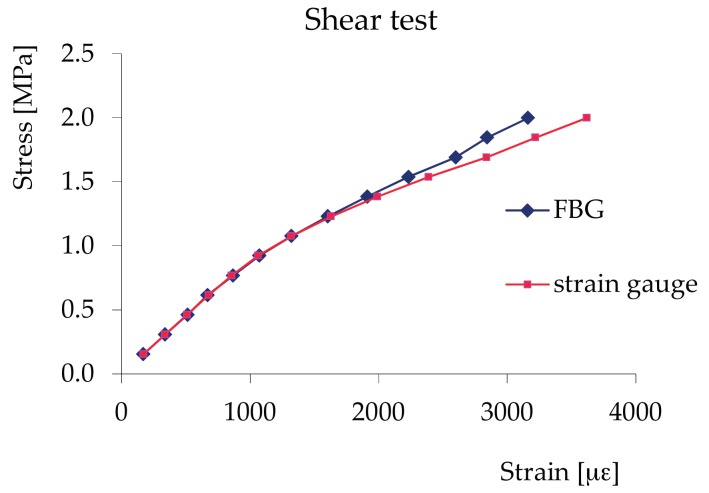
Shear test: stress–strain diagram.

Shear tests—EN 408 2010+A1_July2012 [54] performed on samples with dimensions of 200 × 200 × 46 mm. These tests also showed an excellent correspondence between the two readings up to a stress of approximately 70% of the breaking stress. Beyond this value, there is a non-perfect correspondence (5%) between the measurements with the strain gauge and with the FBG, due to cracking that occurred between the points of application of the two measurement systems, which, in the final part of the test, were under different states of stress (Figure 8).

#### 3.1.2. Verification of Reinforcement/Wood Bonding

**Figure 9:**
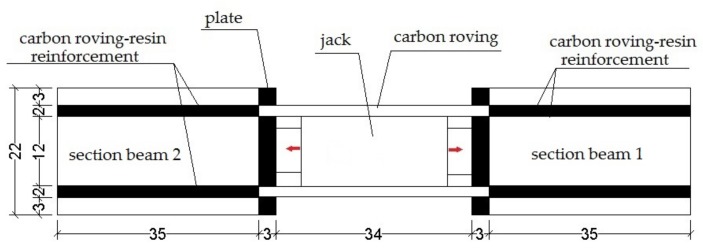
Scheme of the wood-reinforcement adhesion test.

For these tests, samples were prepared consisting of two sections of fir wood beams, each having a length of 350 mm and cross section of 180 × 220 mm. The two wooden beam sections were reinforced with a continuous composite profile consisting of epoxy resin and layers of carbon roving in fibres with a length of 4‰ of the section, inserted inside a cut and sealed with epoxy resin (Figure 9).

A jack was placed between the two beam sections, on one of the ends of each beam, so as to exert traction on the reinforcement. Inductive displacement transducers were applied on the two ends in contact with the jack, in order to measure the relative displacement between the faces of the two beam sections. The tests were monitored with two FBG sensors in series, placed at a distance of 6 cm from the jack load surface: the first embedded in the inner resin layer, the second in contact with the wood in a small incision at the bottom of the milling.

**Figure 10:**
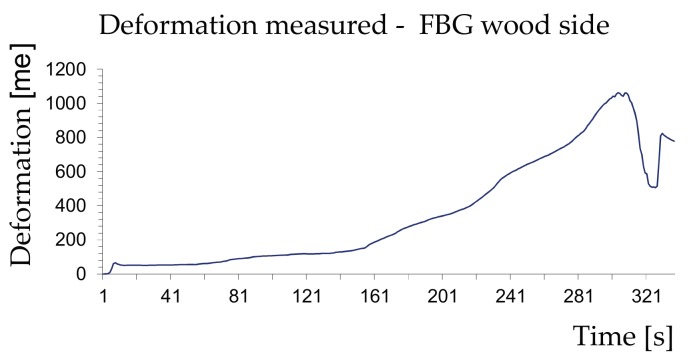
Deformations measured by the FBG in contact with the wood.

**Figure 11:**
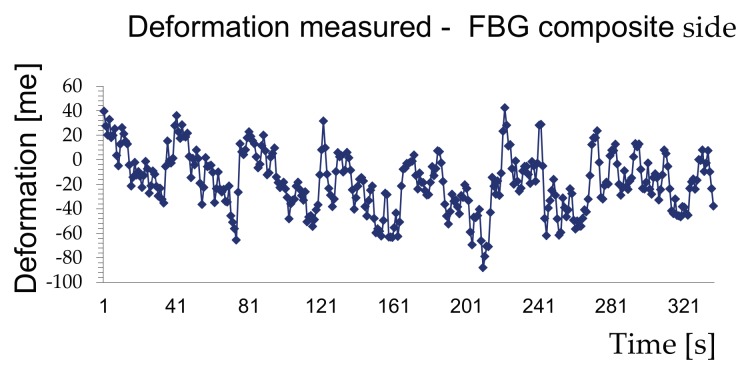
Deformations measured by the FBG in the composite.

The diagrams in Figure 10 and Figure 11 show a typical time-history of the deformations measured during the tests for an acquisition time of 337 s.

The measurements made showed that the samples reached collapse due to the exceeding of the wood’s strength with a tangential stress exceeding 4.3 MPa and sliding within a range of 4–5 mm. It can also be stated that the strength offered by the wood/reinforcement interface is greater than that of wood failure, which demonstrates the excellent effectiveness of the FRP reinforcement technique.

The behaviour of the composite shown in Figure 11 indicates that the reinforcement takes on loading–unloading phases during the test and does not follow the deformation of the wood. This is because sliding planes have been created between the layers of the reinforcement, which was made manually and not industrially.

### 3.2. On-Site Tests on Reinforced Wooden Beams

The measurement methodology based on the application of FBG sensors was used to carry out efficient monitoring of existing wooden beams reinforced with carbon fibre-reinforced polymer (CFRP) after seismic damage.

**Figure 12:**
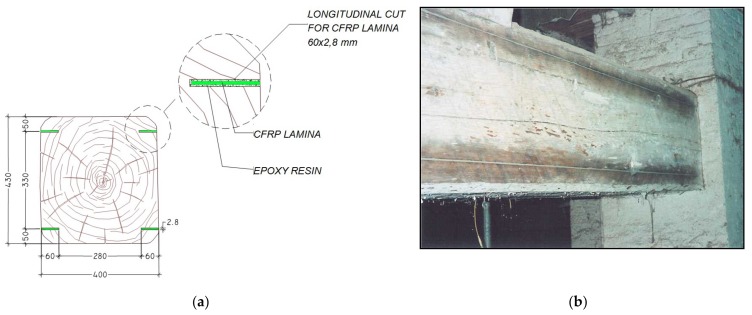
(**a**) The beam section reinforced with CFRP laminae; (**b**) a picture of the beam after the reinforcement (dimensions in millimetres).

The structure considered was a fibre-reinforced floor of a building located in an ancient town near Perugia in Italy and used in the past as farm storage house. The floor is made from a double series of long wooden beams with overlying wooden joists and flat bricks. The floor had to be reinforced for strength and deflection to support heavy loads, while preserving the historic characteristics of the floor. For this reason, the designers decided to apply four CFRP laminae inside the wooden beams, two near the extrados and the other two near the intrados [55]. These laminae, which have a section of 60 × 2.8 mm, were inserted inside cuts made in the beam along the axial direction and then glued into the beam with epoxy resin (Figure 12a). The picture of the consolidated beam in Figure 12b shows minimal intrusivity. Many experimental tests were performed on the primary and secondary beams, before and after consolidation, under the same load conditions. As excitation system—a mass of 200 kg free falling onto the middle of the beam from a height of 1.6 m was used. This excitation type is often used in civil construction investigations, on site.

**Figure 13:**
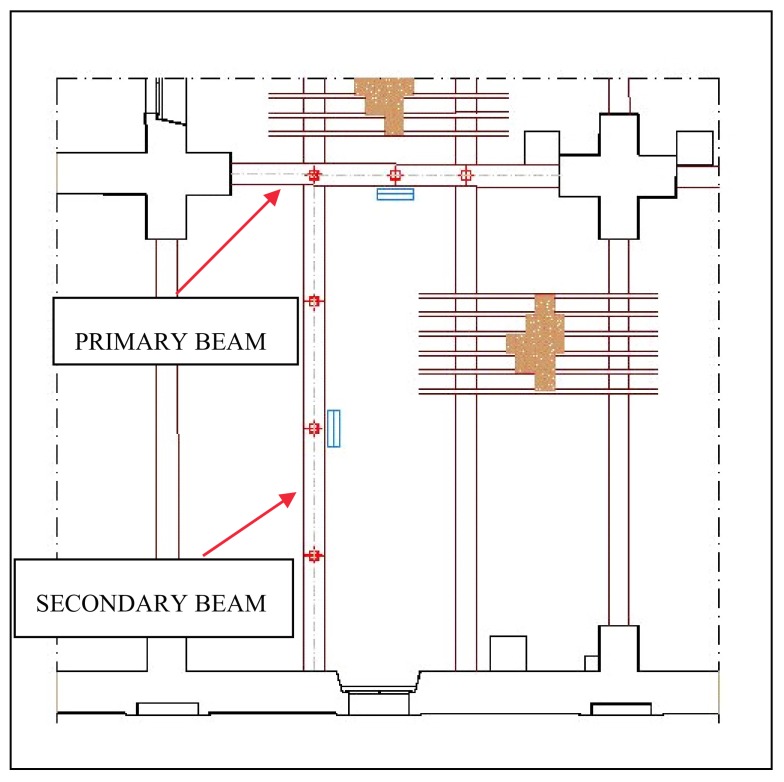
Measurement points: 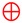 vibrometer and 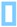 fibre Bragg sensor.

To measure the response of the structure before reinforcement, two fibre Bragg grating sensors were glued at the extrados and the intrados of the beam’s middle section. After reinforcement was applied, other fibre Bragg grating sensors were applied on the two laminae of the right side of the section. The temperature influence was not considered because significant changes in temperature didn’t take place during the tests, and then it was not necessary to use compensation techniques. The laser Doppler vibrometer was used to perform measurements of the structure response at the middle section and at 1/4 and 3/4 of the beam length. The location of the measurement points is shown in Figure 13. Many tests were performed for each condition, before and after reinforcement.

In the following, the most interesting results were seen on the secondary beam because the effect is greater on this beam than on the primary beam, since the second beam is longer (Figure 13). Typically, the tests before reinforcement yielded the following strain variation Δ*ε* due to the impulsive load: −40 µε at the beam extrados and +70 µε at the beam intrados. After reinforcement, the strain variation for the same load was typically −44 µε at the beam extrados, −31 µε at the upper lamina, +48 µε at the lower lamina and +60 µε at the beam intrados. The uncertainty of these results was estimated by standard deviation of results from at least ten measurements below 3 µε. The strain variation at the beam intrados decreases following reinforcement, due to the help provided by the CFRP laminae in supporting the load applied.

**Figure 14:**
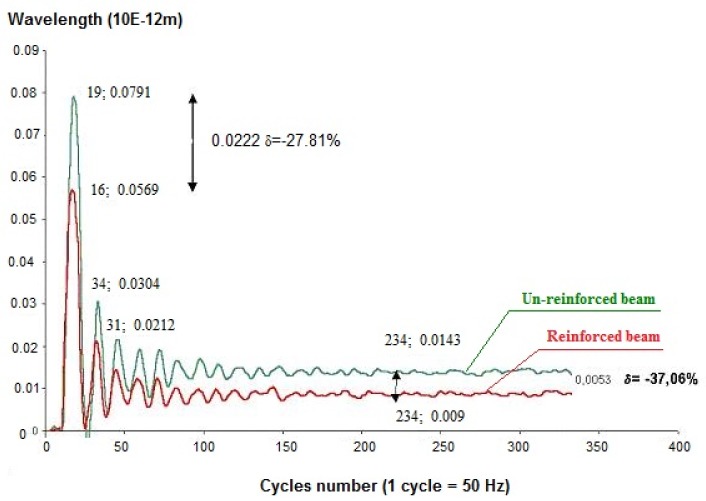
Comparison between the response of the reinforced and un-reinforced beam recorded at the intrados.

The strain reduction can be assessed comparing the signals given by the FBG sensors as shown in Figure 14, which shows the diagrams of the wavelength recorded before and after reinforcement. The difference between the maximum wavelength values illustrated in the figure corresponds to an overall beam deformation reduction, for the same load, of about 27%. It is also possible to observe a significant improvement in the damping coefficient. Similar results were obtained for all the tests, and also for the primary beam.

The values measured using the FBG sensors applied on the CFRP laminae clearly demonstrate the efficiency of the structural connection between the wood and the CFRP laminae, obtained with the structural reinforcement. The two diagrams don’t return to zero due to the residual deformation of the material, greater in the case of an un-reinforced beam.

#### Laser Doppler Vibrometer and Results Validation

The same considerations can be obtained by analysing the laser vibrometer measurements, thus validating the experimental tool based on the FBG measurement techniques. Indeed, the instantaneous vibration velocity value “v” (component along the incident laser beam) of the Laser Doppler Vibrometry is a well-established measurement technique. The measurement principle is based on the Doppler effect that occurs when a laser beam is back-reflected from a vibrating surface, changing the back-reflected light frequency of Δf = 2v/*λ*, with *λ* being the wavelength of the incident laser beam. An interferometer inside the optical head of these instruments gives as output an electrical signal of frequency Δf. An electronic demodulator converts the frequency Δf into a proportional voltage output that can be acquired with a spectrum analyser to obtain, without any contact with the structure, time histories or spectra of its surface vibration velocity. This measurement technique has been successfully applied, for example, for very small dynamic displacement measurements of bridge beams loaded with a single car moving on a road over the bridge [56].

**Figure 15:**
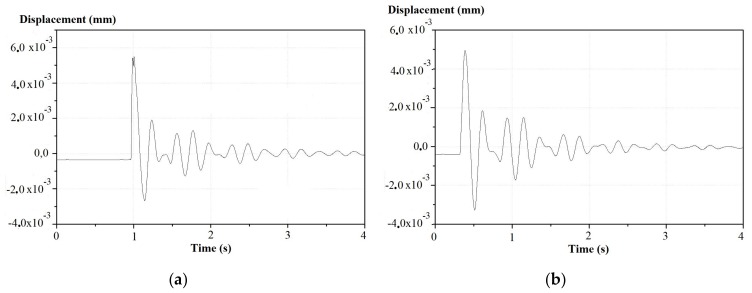
Displacement-time at the intrados middle section in the (**a**) un-reinforced and (**b**) reinforced beam.

**Figure 16:**
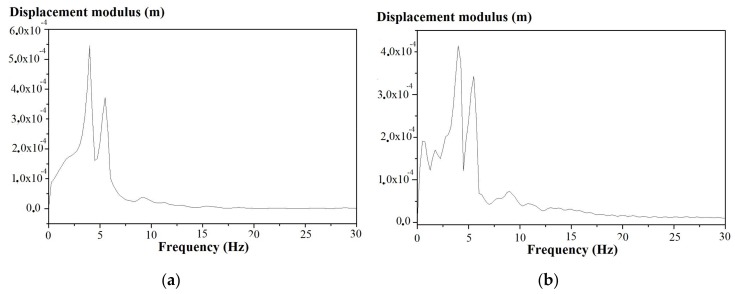
Laser Doppler vibrometer signal spectrum at the middle section intrados in the (**a**) un-reinforced and (**b**) reinforced beam.

The displacements of the wooden beams measured by the laser vibrometer are illustrated in Figure 15a,b, before and after reinforcement in the middle section at the beam extrados. The effect of the reinforcement is not seen in the time domain, i.e., it does not change the amplitudes of the deformations, but only acts on the damping index. Figure 16a,b show the respective laser Doppler vibrometer signal spectra. The peak displacement values recorded are 5.67 mm without and 5.17 mm with reinforcement, respectively, and can be used to obtain similar conclusions to these obtained from the FBG measurement results, e.g., in terms of damping. 

The results obtained by both the laser vibrometer and by FBG sensors prompt interesting considerations about the dynamic behaviour of the beams before and after consolidation. Describing the dynamic behaviour of the beams by using a simple one degree of freedom model, the damping can be evaluated by the expression:

*ξ* = ln(x_1_/x_n_)/(2*π*n), (21)

where “n” is the number of cycles considered and x_1_ and x_n_ are the amplitude corresponding, respectively, to the first and the nth cycles. The measured response of the beam before and after reinforcement allows one to evaluate the mean value of the damping using both the laser vibrometer and the FBG sensor output for the two beams. The results obtained are given in Table 2. It can be observed that the computed values from the vibrometer and from the FBG sensor are very close. Furthermore, the table shows that, in both beams, damping increases after reinforcement (in accordance with Figure 14), and the increase is greater in the secondary beam than in the primary beam. It is highlighted that the vibrational behaviour of the two beams depends on many factors including the characteristic of the wood that is not homogeneous (presence of knots, direction of the grain, density) and the type of constraints (the primary beam is embedded in the masonry while the secondary beam is constrained to the main beam).

## 4. Conclusions

FBG sensors were proposed for the on-site monitoring of wooden beams. A historical building reinforced after an earthquake was analysed using FBG sensors, which were applied also inside the CFRP materials used for restoration and that provided an excellent compromise between reliability and minimal intrusivity. The sensors were successfully installed and allowed the obtaining of strain signals considered very useful for efficient monitoring of the wooden structures tested, after having performed preliminary tests and some theoretical analyses to define optimal installation conditions and techniques.

The results were validated comparing them with laser Doppler measurements of the structure response during the same tests. The results of the comparison make it possible to ensure the reliability of the measurement techniques proposed for effective structure monitoring and to demonstrate the effects of the reinforcement, using FBG sensors, as other techniques.

In this regard, a preliminary analysis of optical fibre-adhesive behaviour was performed and a series of tests were carried out that demonstrated that the measurements made by the glued sensors are increasingly more reliable as the stiffness of the glue increases, so that the adhesive is able to transmit perfectly to the optical fibre the deformations of the material support to which it is bonded. The tests provided very useful information on the bonding behaviour, especially in regards to the bonding thickness and the bonding length.

The measurement technique is non-invasive, and is also suitable for applications on buildings of historic-artistic value. It can therefore be used in the preservation of the historic-artistic heritage for monitoring critical behaviours and for evaluating the effectiveness of the interventions proposed and the improvement achieved.

## Figures and Tables

**Table 1:** FBG and interrogation system properties.

FBG Properties		Micron Optics FBG Interrogation System Properties	
FBG Length	10 mm	Acquisition frequency	1000 Hz
Coating in FBG length	no	Wavelength range	160 nm
Strain limit	5000 με	Wavelength accuracy	1 pm
Strain sensitivity	~1.2 pm/με	Stability	1 pm
Operating temperature range	−40 to 120 C	Repeatability	0.05 pm
Thermal response	~9.9 pm/C	Optical connectors	LC/APC *
Fibre lead length	1 m (±10 cm), each end	Dynamic range	35 dB peak
Fibre type	SMF28-Compatible		
Fibre coating	polyimide		

* LC (Lucent Corporation); APC (Angled Physical Contact).

**Table 2:** Mean damping values estimated by the two measurement techniques.

DAMPING	LASER Doppler Vibrometer		FBG Sensor	
	Before	After	Before	After
secondary beam	0.0851	0.112	0.0850	0.115
primary beam	0.0577	0.061	0.0579	0.0648
